# The first meeting of the European Register of Cystic Echinococcosis (ERCE)

**DOI:** 10.1186/s13071-016-1532-3

**Published:** 2016-04-28

**Authors:** Patrizia Rossi, Francesca Tamarozzi, Fabio Galati, Edoardo Pozio, Okan Akhan, Carmen Michaela Cretu, Kamenna Vutova, Mar Siles-Lucas, Enrico Brunetti, Adriano Casulli, the HERACLES extended network

**Affiliations:** Department of Infectious, Parasitic and Immunomediated Diseases, Istituto Superiore di Sanita, Rome, Italy; European Reference Laboratory for Parasites, Istituto Superiore di Sanita, Rome, Italy; Department of Clinical Surgical Diagnostic and Paediatric Sciences, University of Pavia, Pavia, Italy; WHO Collaborating Centre for the Clinical Management of Cystic Echinococcosis, Pavia, Italy; SIDBAE, Information Technology, Istituto Superiore di Sanita, Rome, Italy; Faculty of Medicine, Hacettepe University, Ankara, Turkey; University of Medicine and Pharmacy, Colentina Clinical Hospital – Parasitology, Bucharest, Romania; Specialised Hospital of Infectious and Parasitic Diseases “Prof. Ivan Kirov”, Department of Infectious, Parasitic and Tropical Diseases, Medical University, Sofia, Bulgaria; Instituto de Recursos Naturales y Agrobiología de Salamanca, CSIC, Salamanca, Spain; Division of Tropical and Infectious Diseases, San Matteo Hospital Foundation, Pavia, Italy

**Keywords:** Cystic echinococcosis, European Register, Public health awareness, Case series, Clinical management, Surveillance

## Abstract

Cystic echinococcosis (CE) is a zoonotic parasitic disease endemic in southern and eastern European countries. The true prevalence of CE is difficult to estimate due to the high proportion of asymptomatic carriers who never seek medical attention and to the underreporting of diagnosed cases, factors which contribute to its neglected status. In an attempt to improve this situation, the European Register of Cystic Echinococcosis (ERCE), was launched in October 2014 in the context of the HERACLES project. ERCE is a prospective, observational, multicentre register of patients with probable or confirmed CE. The first ERCE meeting was held in November 2015 at the Italian National Institute of Health (Istituto Superiore di Sanita, ISS) in Rome, to bring together CE experts currently involved in the Register activities, to share and discuss experiences, and future developments.

Although the Register is still in its infancy, data collected at the time of writing this report, had outnumbered the total of national cases reported by the European endemic countries and published by the European Centre for Disease Prevention and Control in 2015. This confirms the need for an improved reporting system of CE at the European level. The collection of standardized clinical data and samples is expected to support a more rational, stage-specific approach to clinical management, and to help public authorities harmonize reporting of CE. A better understanding of CE burden in Europe will encourage the planning and implementation of public health policies toward its control.

## Background

Cystic echinococcosis (CE) is a chronic zoonotic infection transmitted to humans through the ingestion of eggs of the tapeworm *Echinococcus granulosus* (*sensu lato*) (*s.l.*) shed in the faeces of dogs harbouring the parasite in the intestine. Humans are accidental intermediate hosts, where the parasite can develop into fluid-filled cysts (metacestode stage) located mainly in the liver and lungs [1].

CE has a worldwide distribution, and affects mainly individuals in poor rural communities in underserved livestock-breeding areas. In 2010, this disease was included in the list of 17 Neglected Tropical Diseases (NTD) prioritized for interventions by the World Health Organization (WHO; http://www.who.int/neglected_diseases/diseases/en/). Current estimates indicate a prevalence of 2–3 million global cases and incidence of around 200,000 new detected infections/year [2]. The burden of disease was estimated to range between 1 and 3.6 million Disability Adjusted Life Years (DALYs), with an annual cost of 3 billion US$ accounting for human treatment and livestock production losses [3, 4].

However, the real prevalence, incidence and burden of CE are difficult to estimate. This is due to the patchy distribution of CE within transmission areas, the high proportion of asymptomatic infected individuals and symptomatic patients living in resource-poor areas with logistical and/or economic constraints, who never reach medical attention, and the unknown effect of underreporting of diagnosed cases [5–7]. This last aspect is primarily due to lack of CE mandatory notification overall. In addition, diagnosis at the species level, differentiating cystic from alveolar echinococcosis in the majority of Member States is limited. In fact, in Europe the origin of available data on human CE is very heterogeneous, and most data are patchy and collected from hospital records. Therefore, patients receiving medical attention in an outpatient setting are not registered in hospital records and are generally not captured.

**Fig. 1:**
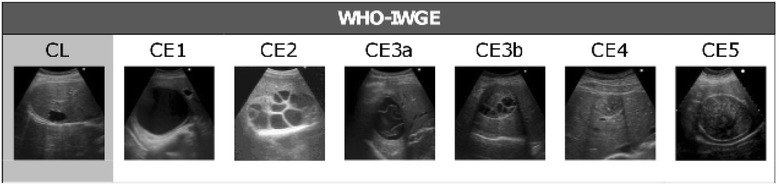
Schematic representation of the ultrasound classification of hepatic CE according to WHO-IWGE. CE1, fluid filled active unilocular cyst; CE2, active cyst with daughter vesicles; CE3a, transitional cyst with detached endocyst; CE3b, daughter vesicles in a solid matrix [although classified as transitional, this cyst stage is biologically active]; CE4, inactive cyst with solid matrix; CE5, inactive cyst with solid matrix and calcified walls

The difficulties in assessing the burden of disease are compounded by the peculiar clinical characteristics of CE, which impact not only the diagnosis but also the treatment and therefore, the costs associated with the disease. In humans, echinococcal cysts grow slowly, passing through different stages, as described by the ultrasound classification issued by the WHO Informal Working Group on Echinococcosis (IWGE) (Fig. 1; [8]). The WHO-IWGE also indicated that for liver locations, different stages should be managed with different approaches (chosen among surgery, percutaneous treatments, medical treatment, watch and wait). However prospective clinical trials to compare these options are lacking. In addition, this stage-specific approach is not widely practiced and the management of the disease is often inappropriate. These conditions make it difficult to issue evidence-based guidelines for the management of CE and expose patients and health systems to unnecessary treatments and costs [7, 9, 10].

Finally, CE is a preventable infection, although elimination is difficult to achieve and, using mathematical models, is estimated to take between 10 and over 25 years of sustained efforts depending on the combination of interventions implemented and their coverage [11, 12]. Technological improvements in the genetic characterization of strains and vaccination against *E. granulosus* in animals could increase the efficiency of control programs, and make them shorter [13]. It is therefore important to acquire a better understanding of human epidemiology, burden of infection and clinical management strategies, to optimize the control approach.

In 2013, HERACLES (Human cystic Echinococcosis ReseArch in CentraL and Eastern Societies) a translational collaborative project funded by the European Commission under the Seventh Framework Programme (http://www.Heracles-fp7.eu/) was established. The main aims of HERACLES are to assess the prevalence of human abdominal CE through extended ultrasound surveys in endemic rural areas of Bulgaria, Romania and Turkey, the endemic countries targeted for screening, to promote a stage-specific approach to clinical management through education activities directed at health care workers, and to improve serological and molecular diagnostic tools. Within HERACLES, the implementation of an international register of human CE to provide a tool to record new cases, their treatment, and the evolution of individual cysts over time, was identified as essential in order to improve public awareness and clinical management of CE. Indeed, while improvement of notification requirements, improved surveillance, and research to clarify the basis of underreporting are essential requirements, they need to be carried out at the government/central level. The implementation of an international register based on voluntary adhesion of centres where CE patients are managed, is an essential step to provide data to support such actions. Additionally, within the HERACLES project, the Echino-Biobank was created to host patients’ and parasite samples that can be used in multicenter analyses approaches for serological and genetic studies, which often are underpowered when only samples from individual centres are used.

ERCE is the first CE register, whereas the existent European Echinococcosis Registry (EurEchinoReg) is exclusively dedicated to the surveillance and reporting of patients with alveolar echinococcosis, and has a different structure [14].

## The first meeting on ERCE

**Fig. 2:**
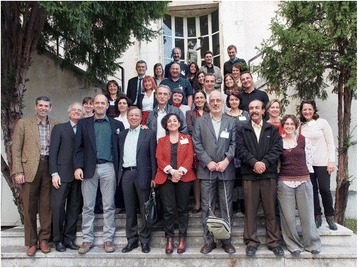
The participants of the European Register of Cystic Echinococcosis (ERCE). Representatives of the centres from 16 European and Asian countries (Albania, Austria, Bulgaria, France, Georgia, Greece, Hungary, Iran, Italy, Palestine, Poland, Romania, Serbia, The Netherlands, Spain and Turkey) at the official launch meeting on ERCE. The meeting was held at the Italian National Institute of Health (Istituto Superiore di Sanita, ISS) in Rome, Italy, between 12 and 13 November 2015

The first meeting on ERCE was held at the Italian National Institute of Health (Istituto Superiore di Sanita, ISS) in Rome, between 12 and 13 November 2015, to bring together both ERCE active participants and those interested in joining the Register and to share and discuss experiences, issues and future developments. Forty-four participants with expertise in the management of *Echinococcus*/echinococcosis from 16 countries (Albania, Austria, Bulgaria, France, Georgia, Greece, Hungary, Iran, Italy, Palestine, Poland, Romania, Serbia, Spain, The Netherlands and Turkey) participated in the meeting (Fig. 2). The meeting was also supported by the Department of Control of Neglected Tropical Diseases (WHO, headquarters Geneva), a member of the External Advisory Board of the HERACLES project. During the meeting, the ERCE and HERACLES activities carried out to date were presented, as well as overviews on the epidemiology of *E. granulosus* infection in animals and humans, stage-specific approach to treatment of CE, percutaneous treatment of CE, and immunodiagnostic tests for CE diagnosis [7, 15–18]. On the second day of the meeting, training on the use of the software developed for the Register was offered, employing an online trial version of ERCE. Each participant had the chance to work with the Register, by entering data of mock patients. During the meeting, the core topics on ERCE, described below, were illustrated.

### ERCE structure

ERCE is a single multicentre database located within the secured IT network of ISS, in Rome, where patient data, including demographic information, CE-related clinical data (unequivocal identification of single cysts, cyst characteristics and treatment administered), and biological samples collected are recorded (Table 1). Biological samples (serum, plasma, cyst-derived material, etc.) with respective clinical information provided by voluntarily adhering centres are stored at the Echino-Biobank, established in Salamanca, Spain (IRNASA-CSIC; Instituto de Recursos Naturales y Agrobiologia de Salamanca – Consejo Superior de Investigaciones Cientificas) within the HERACLES project. This repository of samples is registered as an official biobank collection (registration number C.0003432) and is managed according to the European standards for biobanking (Law 1716/201), including anonymization of samples through their (-80 °C) storage in 2D-barcoded tubes that can be scanned and automatically linked to the corresponding clinical data in ERCE.

**Table 1:** Schematic overview of the European Register of Cystic Echinococcosis (ERCE) features

Patients enrolled in ERCE
− with confirmed or probable CE (direct evidence of infection, imaging-based diagnosis alone or associated with positive serology) − in- and out-patients − all ages and both sexes − first visit or follow-up visit
Data recorded for each patient
− personal data: year of CE first diagnosis and history of treatments − clinical data: cyst(s) localization, size and stage, administered treatment(s) − data on the biological samples collected (if any) − ERCE complies with Italian (Italian Personal Data Protection Code n. 196, 2003) and European (2000/C 364/01, and Directive 95/46/EC) regulations on the protection and use of personal data − two informed consent forms must be signed by patients at initial registration to allow: * their data to be recorded in the Register * their biological samples to be shipped to the Echino-Biobank
ERCE structure
− single multicenter database located within the secured IT network of the Italian National Institute of Health (Istituto Superiore di Sanità, ISS) in Rome − organized in sheets where patient data are recorded − each registered patient is automatically given a unique ERCE ID code − data are uncoupled and anonymized
ERCE users
− physicians working in health centers where patients with CE are managed − join ERCE network voluntarily − are provided with personal credentials to login to the register − different roles are envisaged: * the “person in charge” in each centre enters patients’ data * the “supervisor” in each centre can read only data of his/her centre * the Register “coordinator” has access to and can download data from all national centres
Requirements to join ERCE
− to be a physician working in centers where CE patients (in- and out-patients) are visited − to get the approval from the Ethics Committee (EC) of each centre/country involved (although the implementation of the Register is only observational and does not involve clinical experimentation)
Ownership of data
− data from individual centres belongs to the individual centres themselves − the coordinator can only use cumulative data for periodic presentations on the progress of ERCE − publication of data requires the consent of the individual centres

### Ethical approval

**Fig. 3:**
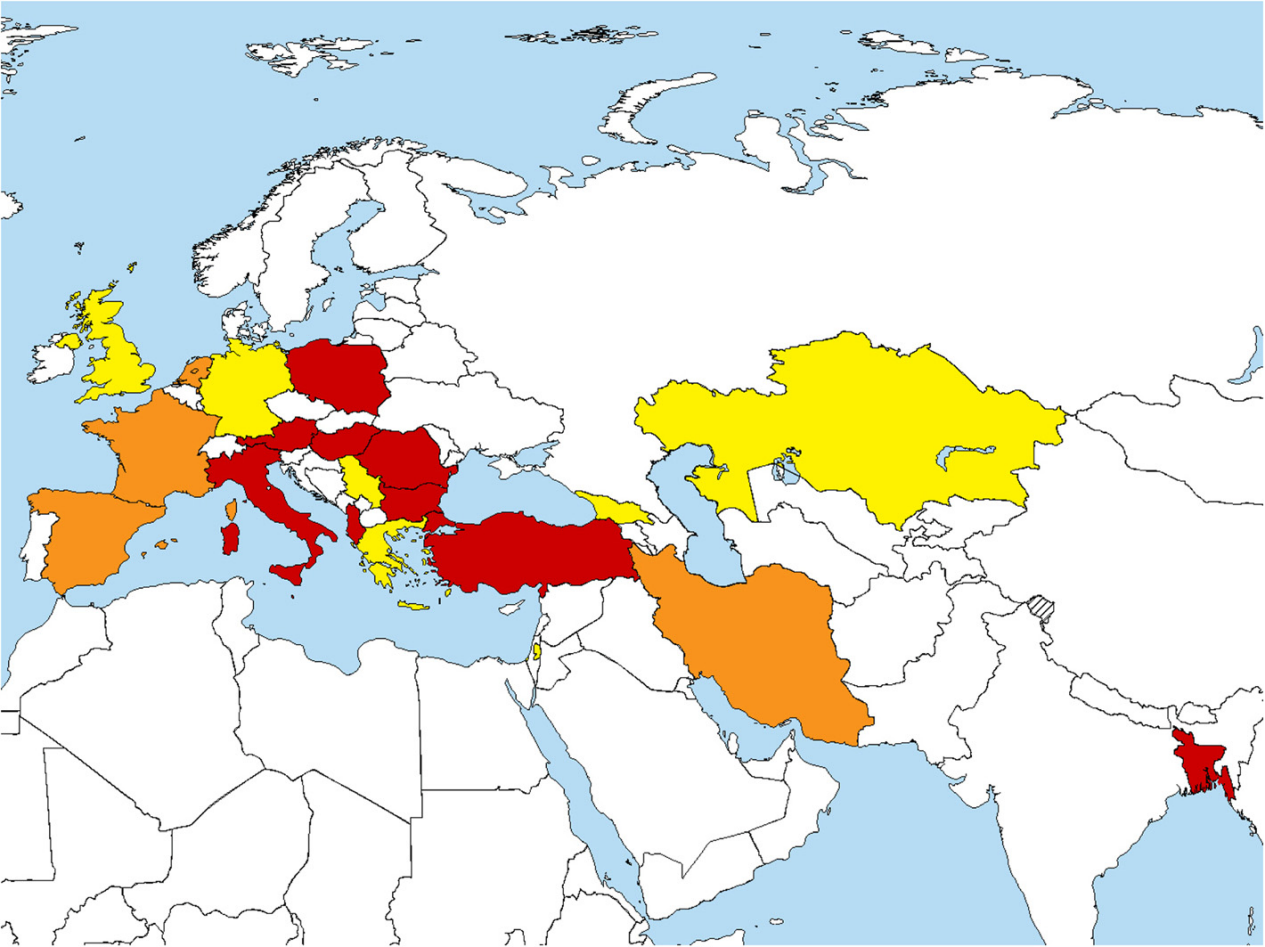
Countries currently involved in the European Register of Cystic Echinococcosis (ERCE). In *red*: countries with ERCE affiliated centres that received the clearance from ethics committee and enrolled patients so far. *Orange*: countries with ERCE affiliated centres that received only the clearance from ethics committee. *Yellow*: countries interested or in the process of affiliation to ERCE. More information available at: http://www.heracles-fp7.eu/erce.html

ERCE was developed by re-structuring and expanding at international level the Italian Register of CE (RIEC) implemented in 2012 [19]. RIEC was approved by the ISS Ethics Committee (Ns Prot. CE/12/347 of May 7th, 2012), which extended its agreement to ERCE (Prot. PRE-C-915/14 of November 25th, 2014). Since its inception, more than 845 patients from 24 affiliated centres in nine countries (Albania, Austria, Bangladesh, Bulgaria, Hungary, Italy, Poland, Romania and Turkey) have been enrolled (Fig. 3). CE patients identified during the ultrasound surveys carried out in Bulgaria, Romania and Turkey in 2014 and 2015 as part of the HERACLES project are also listed in ERCE, and their biological samples are included in the Echino-Biobank repository. The Register has recently been extended to extra-European countries.

### Aims of ERCE

The European Register of Cystic Echinococcosis (ERCE; http://www.heracles-fp7.eu/erce.html) was created with the aim of providing a more appropriate tool for the surveillance of human CE, built to take into account the peculiar features of the infection such as chronicity and case management often carried out in an outpatient setting, and to establish a prospective case retrieval to evaluate the spontaneous or treatment-related evolution of individual cysts. This will offer the opportunity to study prospectively and systematically a stage-specific approach to treatment, rate of adverse reactions, relapse rate and costs of CE infection. Data collected in ERCE will allow national authorities and international agencies such as the European Centre for Disease Prevention and Control (ECDC) to acknowledge the magnitude of the problem by reporting cases otherwise not captured by systems such as hospital discharge records. Furthermore, it will offer a prototype register structure to harmonize data collection and reporting of CE including data on the pivotal clinical features of the infection, to ultimately encourage and support with data the planning and implementation of public health policies toward its management and control.

ERCE addresses the research questions concerning the number of CE patients visiting each affiliated centre, their geographical origin, their clinical management, and the spontaneous or treatment-induced evolution of the cysts over time. The collection of such data in a consistent, harmonized way will help reduce the difficulties in clinical decision-making caused by the lack of prospective studies, which are not affordable at the current rate of funding for research on CE [3]. It is widely known that the majority of clinical studies on CE are retrospective, and underpowered by the small number of homogeneous samples that each centre has access to. The Echino-Biobank, a wide collection of biological samples with reliable clinical data from CE patients, will support this action.

### Usefulness of ERCE for individual participating centers

Both individual centers and the scientific community at large will benefit from the data stored in the ERCE database. The unique ERCE ID assigned to each patient, together with the possibility of updating patient records at each follow-up visit, allows the tracking of new diagnoses, patients referred by other centres, compliance and lost-to-follow-up rates, demographic structure and geographical origin of the patient population of each centre. From a clinical perspective, the unequivocal identification of each cyst in the Register facilitates the follow-up and clinical evaluation of the evolution of each cyst, either spontaneously or through treatment induction allowing a better evaluation of the stage-specific outcome of each clinical management approach. Finally, by participating in the Echino-Biobank, centres involved in research activities may benefit from accessing a wide panel of well-characterized sera and other biological material, performing powerful studies, otherwise difficult to carry out by using a limited number of samples from single centres. Access to the samples hosted in the Echino-Biobank will be evaluated in a project-based frame by the ethical and scientific committees of the biobank.

### Critical issues

The voluntary nature of adhesion to ERCE and the data recording based on the goodwill of single clinicians will limit the use of data from ERCE to make estimates on prevalence and incidence of infection. However, altogether, the information collected prospectively and in a consistent way within ERCE will support decisions regarding the implementation of control programs, of better designed reporting systems, and the issue of case management recommendations. A critical evaluation of data quality will be carried out yearly, and a comprehensive evaluation of the usefulness of the Register will be carried out after five years.

The sustainability of the Register over time after the end of HERACLES project is guaranteed, since Registers at ISS are officially considered as an institutional commitment currently maintained by dedicated permanent staff for which no funding is needed. The Echino-BioBank will be sustained over time by the European Union Reference Laboratory for Parasites (http://www.iss.it/crlp/).

### The HERACLES extended network

This international network was recently created within the framework of HERACLES collaborative project, and it extends the capacity of the core partners of the project (http://www.heracles-fp7.eu/interactive_map.html). The HERACLES extended Network is currently represented by more than 40 centres from 23 European and Asian nations, most of whom are participating in ERCE. The network includes mostly clinicians dealing with diagnosis and clinical management of CE, but also experts on CE epidemiology, diagnosis, molecular characterization and control, providing advice and support. The aim of this network is to support core activities within HERACLES, such as enrolling patients in ERCE, providing parasitic and human samples to the Echino-Biobank according to a standardized protocol, and cooperating in research studies on serology and molecular epidemiology of CE. The ultimate goal is to promote health equity on CE providing common background, tools and access to biological and medical knowledge for the centres involved in the HERACLES extended Network.

## Conclusions

The true number of CE cases in Europe is unknown mainly due to the lack of mandatory notification requirement. In 2015 the ECDC reported 801 confirmed human cases of ‘echinococcosis’ from 14 Member States, but the differentiation between cystic and alveolar echinococcosis was not available [20]. As stressed by the European Food Safety Authority [21], a re-evaluation of the case definition for ‘echinococcosis’ in the current EU decision 2012/506/EU, differentiating these two diseases, will be crucial in order to collect real epidemiological and clinical data to manage and track these infections.

The collection of essential clinical data using a standardized approach will provide valuable information on the clinical history of the infection in populations living in endemic areas. The experience from ERCE will enable governments, the European Commission and related European agencies such as ECDC, to harmonize data collection, monitoring and reporting of CE, according to EU legislation (Directive 2003/99/CE of the European Parliament). Additionally, samples hosted in the Echino-Biobank will support evidence-based studies in the diagnosis and follow-up of CE patients.

We encourage other researchers and clinicians around the world to participate in ERCE, and help the many patients suffering from a complex and chronic disease that has been under the radar for too long.
